# Estimating Intervention Effects in a Complex Multi-Level Smoking Prevention Study

**DOI:** 10.3390/ijerph6020463

**Published:** 2009-02-03

**Authors:** Milena Falcaro, Andrew C. Povey, Anne Fielder, Elizabeth Nahit, Andrew Pickles

**Affiliations:** 1 Biostatistics Group, School of Community-Based Medicine, University of Manchester, UK; E-Mail: andrew.pickles@manchester.ac.uk (A. P.); 2 Occupational and Environmental Health Research Group, University of Manchester, UK; E-Mails: andy.povey@manchester.ac.uk (A. C. P.); annfielder@hotmail.com (A. F.); LNahit@ccm.ac.uk (E. N.)

**Keywords:** Instrumental variables, multi-level intervention study, non-compliance, treatment effect

## Abstract

This paper illustrates how to estimate cumulative and non-cumulative treatment effects in a complex school-based smoking intervention study. The Instrumental Variable method is used to tackle non-compliance and measurement error for a range of treatment exposure measures (binary, ordinal and continuous) in the presence of clustering and dropout. The results are compared to more routine analyses. The empirical findings from this study provide little encouragement for believing that poorly resourced school-based interventions can bring about substantial long-lasting reductions in smoking behaviour but that novel components such as a computer game might have some short-term effect.

## 1. Introduction

This paper presents an analysis of the UK component of the European Smoking Prevention Framework Approach (ESFA) study, illustrating and explaining how several difficulties that are commonly encountered in intervention studies may be tackled.

The ESFA study was a large cluster-randomised study aiming to reduce smoking among adolescents. In such studies clusters of observations (in our case, pupils in schools) are sampled and randomly assigned to a control or an experimental group and only the subjects belonging to the latter receive the new treatment. Any difference in the outcome variable between the two groups is then seen as the effect of the treatment. Unfortunately, many intervention programs are frequently complicated by non-compliance, i.e. a departure from the study protocol caused by subjects not following or being unable to follow the treatment regime to which they were assigned. Since it makes little sense to look for an intervention effect among those who received no treatment, researchers are then tempted to use the comparison of the outcome for groups defined by those who received and those who did not receive the treatment. However, since non-compliers are invariably systematically different from compliers, this comparison is likely to be biased.

Further complexities may arise when, as in the study described in this paper, the treatment is composed of several elements and this set of treatment elements is randomized as a set not to individuals but to, for example, schools. It may then not be possible to establish with certainty to which treatment elements each subject was exposed.

Moreover, studies typically may have a longitudinal design, requiring not only the linking of responses over time and proper accounting for the lack of independence between observations but also tackling the inevitable sample attrition, which may arise at different levels. For example, if we observe students over several years, we may have missing values because the single student drops out or because the school withdraws from the study and consequently all the students of that school are lost. Furthermore, the pattern of missing data cannot always be assumed to be monotone. There may be schools that withdraw from the study but decide to re-enter later on. With longitudinal studies one may also be interested in investigating the cumulative effect of the treatment over time.

This paper attempts to address these practical problems. In section 2 we describe the ESFA study and its complexities. In section 3 we illustrate how we can estimate cumulative and non-cumulative treatment effects for a range of treatment exposure measures (binary, ordinal and continuous) while adjusting for non-compliance, clustering and drop-out. In section 4 the analysis and results of the ESFA study are described. Lastly, section 5 contains some final comments.

## 2. The ESFA Study

The ESFA Project was set up with the aim of implementing and evaluating the impact of various smoking prevention activities in six European countries. The target population was 12 to 16-year-old adolescents in secondary education. This trial was novel in attempting to promote change through an intervention involving individual, class, school, family and community based activities. We here consider the UK component of the study.

### 2.1. Sample Design and Interventions

At baseline 42 schools were selected and assigned to either an experimental or a control group and a total of 6,626 students entered the study. Control schools were asked not to carry out any new smoking prevention activities, whereas experimental schools were encouraged to implement an intervention package over three consecutive years (1999, 2000 and 2001). The interventions were spread over the whole intervention period and consisted of a set of activities, among which were lessons, leaflets and a computer game, aimed to awaken students to the hazards of smoking and to warn them about tobacco adverting techniques. More details about the study are available [1].

### 2.2. Questionnaires, Compliance and Characterizing Treatment Exposure

Each of the participating schools designated a contact person to supervise the questionnaire management and to facilitate communications between the school and the ESFA team. Treatment was delivered via the teachers. Pupils were asked to fill in a questionnaire at the baseline (1998) and in each of the subsequent intervention years. Both teachers and school contact persons were invited to complete a questionnaire with questions intended to assess compliance. Unfortunately, attempts to obtain teacher reports of intervention delivery and acceptability largely failed, making it impossible to determine with certainty which part of the intervention package pupils actually received. We were thus left with indications of treatment exposure from the students’ self-reporting and from the school contact persons’ questionnaires. On the basis of the students’ recalled exposure to elements of the program, it became clear that the various components were neither received by all pupils in intervention schools nor were they exclusive to these schools. Moreover, within each school there was very substantial disagreement among pupils on exposure even where that exposure was likely to be a shared experience. The differences between the control and the experimental schools in terms of students’ recall of the intervention were mostly quite modest, apart from that related to the computer game. Among other measures, the school contact person reported about the number of lessons carried out by the school on smoking prevention. With only a single reporter we have little scope for assessing its reliability but in face validity terms this seemed to be a good indicator of the overall amount of treatment exposure. In this paper we will therefore focus on two measures of compliance: exposure to the computer game as reported by the students and the number of smoking-prevention lessons carried out by the school as reported by the school contact person.

### 2.3. Outcome Variable

In common with many studies, we focused on students being or becoming regular smokers, defined on the basis of self-report average smoking of at least once a week [2,3]. Adolescent self-report on smoking appears to be reliable provided that anonymity can be assured [4,5]. In the ESFA study strict confidentiality was promised to the pupils before completion of the questionnaire.

## 3. Analysis Issues in Complex Multi-Level Intervention Studies

The Introduction summarized how the estimation of treatment effects are often complicated by issues such as non-compliance, missing values and correlation between observations. In this section we describe how these problems can be tackled.

### 3.1. Estimating Treatment Effects in the Presence of Non-Compliance

Several methods have been proposed for the estimation of treatment effects. Assuming random assignment, perfect compliance and no missing data the treatment effect can be estimated by the difference in the mean outcome between those in the control arm and those in the intervention arm. This is the gold standard Intention-To-Treat (ITT) estimator of intervention efficacy [6]. An ITT analysis is usually straightforward since it requires just the fitting of some appropriate regression model with the randomization indicator as the only predictor and it completely ignores whether or not the subject actually received the treatment. However, if there are departures from randomization, this can lead to misleading results [7].

In a so-called As-Treated (ATT) analysis the subjects are analyzed as if they were randomized to the treatment they actually received, regardless of which arm of the trial they were initially allocated to. The treatment effect is thus estimated as the difference in outcome means between those who got the treatment and those who did not. Nevertheless, if those who comply with treatment assignment are systematically different from non-compliers, this comparison is likely to be biased by the presence of confounders [8].

Adjustment for measured confounders is possible by use of a propensity score [9], a measure that summarizes the selective receipt of treatment associated with a set of measured factors and covariates. Undertaking an ATT analysis while matching, covarying or weighting by the propensity score provides a treatment effect adjusted for those confounders [9,10]. To account for selective assignment related to both measured and unmeasured confounders we can use an instrumental variable (IV) approach, much used in econometrics [11,12]. Consider a linear model for the outcome *Y:*

(1) Y=α+β D+ε

where *α* and *β* are parameters, *D* denotes treatment received and *ɛ* is an error term with E(*ɛ*) = 0 and Var(*ɛ*) = *σ*^2^. If there is selective treatment exposure and we do not adjust for all the possible confounders, *D* and *ɛ* are correlated and *D* is said to be endogenous. Any other variable which is not correlated with *ɛ* is referred to as exogenous. In the presence of endogeneity we cannot simply estimate the treatment effect *β* via ordinary regression. A way to overcome this problem is to find an IV, a variable that is correlated with the endogenous treatment variable *D* but uncorrelated with the error term *ɛ*. Informally this can be thought of as basing the estimate of treatment effect on a part of the variation in treatment exposure that is known to be uncorrelated with confounders. However, finding such a variable has proved to be a difficult task in many contexts. In randomized trials the problem is made much simpler since the treatment assignment indicator *W* (*W* = 1 if the subject is assigned to receive the treatment and *W = 0* otherwise) is an obvious IV.

In regression terms the IV approach is conceived in the form of two equations: one for the outcome variable and one for the endogenous treatment variable, namely

(2) $$Y=\alpha_{1}+\beta_{1}\;D+\varepsilon$$

(3) $$D=\alpha_{2}+\beta_{2}\;W+\zeta$$

where *α*’s and *β*’s are parameters, *W* is an IV for *D*, whereas *ɛ* and *ζ* are error terms such as *Cov*(*ɛ*,*ζ*) ≠ 0. In other words, when there is non-compliance we cannot simply estimate the effect of treatment by using equation (1) because there are confounding variables, which often are unmeasured, that influence both the outcome and treatment received. The IV method consists in finding a variable W that is correlated with D and that affects Y only through D and in then fitting the pair of equations (2) and (3).

With a continuous response *Y* the treatment effect *β_1_* can be consistently estimated by a two-step procedure (2SLS). At the first stage *D* is regressed on *W* as well as other exogenous variables, whereas at the second stage *Y* is regressed on the exogenous variables and on *D̂*, the predicted value of the endogenous treatment variable obtained from the previous stage. The standard errors of the second-stage estimates have to be adjusted to account for the two-step estimation. For outcomes requiring non-linear models, such as probit or logistic regression for binary outcomes, the analysis is not so straightforward since this two-step procedure no longer yields a consistent treatment effect estimate [13]. A way to overcome the problem is by using a simultaneous equations framework. For example, if both the outcome *Y* and the endogenous treatment *D* variables are binary, we can use a bivariate probit model [12,14] which consists in a joint estimation of different probit models for *Y* and *D* where the error terms of the 2 models are allowed to be correlated. For outcome and treatment exposure measures of ordinal or mixed types then appropriate models have to be specified in structural equation modelling programs such the Stata procedure *gllamm* [15].

Although the IV method does not require the assumption of no unmeasured confounders, this is gained at the expense of a possible loss of efficiency. This can be extreme if the available IV is only weakly correlated with treatment exposure. Furthermore, if the intervention being trialled involves a combination of several elements, non-compliance may occur at the level of the whole combination (i.e. individuals receive none or all of the elements) or more commonly element by element. The IV approach can be used to test each element in turn; testing the elements simultaneously is more difficult because each element requires a distinct IV and with a single combination protocol there is usually just one random assignment variable available for use as an IV.

With longitudinal intervention studies one may also be interested in the cumulative effect of receiving treatment over time. This means that at time T we aim to estimate the effect of a variable Λ*_T_* representing the total amount of treatment accumulated by the individual up to a time *T, i.e.* $\Lambda_{T}=\sum_{t=1}^{T}\delta_{t}$ where *δ_t_* represents the binary indicator of treatment exposure or the dose of the treatment received at time *t*.

### 3.2. Missing Values and Linking Records

In the ESFA study missing data were common and drop-out occurred at both the individual and school levels. At school level it was straightforward to identify schools that had dropped out, and dropped back in, during the study. However, identifying individual level drop-out was more complicated than expected since it required that respondents always maintain the same identifier at all occasions. Unfortunately, in our study there were a proportion of records that failed to link across time that were most likely due to errors in identifiers rather than student turnover and absences. There were also a smaller number of records that shared identifiers but were clearly different individuals. It is known that the reliability of self-report smoking relies critically on convincing students of confidentiality through assurances of anonymity. It is easy for anonymity to be confused with lack of identifiability. These concerns may have resulted in less attention being given to the consistent use of student identifiers. Before proceeding with the analysis, procedures were therefore developed to check the validity of linked records and to link near identical but formally unlinked records. Nonetheless, while good progress was made with improved linkage enabling the fitting of multilevel longitudinal models, there remained some concern. As a result, we favoured methods of analysis that rested less heavily on formal linkage of individuals over time. We assumed that, while the probability of missing at the individual level might vary with covariates included in the model, it was otherwise constant. We then accounted for the school-level missingness via weighting. This method adjusts for discrepancies between the obtained and the target sample of schools caused by missing data. If for example our sample can be divided in two strata, one with high and the other with low attrition, then compared to participants from the low attrition stratum, each subject that remains from the high attrition stratum is given a larger weight since each one must represent the larger number of similar subjects from that stratum that were lost through attrition. The weights are derived as the inverse of the “sampling” fraction (the proportion with complete data) from each stratum.

In this analysis the weights are defined as the inverse of the relative probability for a school to be in the sample. Logistic regression was used to estimate the probability at each follow-up for each school participating in the study. These school and occasion specific weights then weighted each record in a pseudo-likelihood analysis of treatment effect. While the application of weights may correct for bias in effect estimates their use makes invalid the usual methods for calculating standard errors and other estimates of precision and significance. These must be calculated using weighted scores or bootstrap. Good overviews on the use of sampling weights can be found in [16,17].

### 3.3. Dealing with Multi-Level Longitudinal Data

With multi-level longitudinal data it is necessary to account for the correlation between observations [18,19]. The two main approaches proposed for the analysis of correlated data relate to two broad classes of models, namely random effects or conditional and population-averaged or marginal models. In the former the correlation is typically accounted for by including in the model a cluster-specific random component [20]. In the population-averaged approach the primary focus of interest is the factors influencing the expected or conditional mean of the response variable and the correlation among observations is treated as a nuisance [21,22]. Which approach to use depends mainly on the research question and whether the correlation is treated as a nuisance parameter or a quantity of interest. Moreover, for binary and ordinal outcomes the usual effect estimates derived from population-averaged and random effects models do not estimate the same parameter, the latter estimator being conditional on unobserved random effects and tending to be larger. However, the effect estimate from the random effects model is appropriate to a comparison of two randomly sampled individuals with different treatment exposure that can be found from comparing predicted outcome rates averaged over the random effects distribution and this does provide an estimator comparable to that of the population-averaged approach. In social intervention studies, since the interest lies principally in understanding population-level rather than individual-level effects, the population-averaged framework is more directly suitable, providing the effect estimate of interest without further work. Had our study included richer data in relation to school context that would have allowed a more detailed examination of the factors associated with between school variance and how much of that variance they could explain, or had the study a more specific focus on between school variability in treatment effect, then a random effects approach might have been preferable.

In its simplest implementation the population-averaged method consists in fitting models to the data as if observations were independent and then accounting for the clustering by using a robust estimator for the standard errors [23,24]. Since it does not require us to specify the correct correlation structure among observations, a complete linkage of records over time at the individual level is less necessary. This method also provides appropriate estimates and standard errors where data are weighted.

## 4. Results

The number of schools participating in each of the four years were respectively 22, 18, 16 and 15 intervention schools and 20, 16, 10 and 11 control schools. School dropout was likely to be related to the importance assigned to the topic, implicit evaluations of treatment effectiveness, staffing and the like and thus not entirely random. Therefore, for each of the intervention years we used a logistic regression to model the probability for school study participation as a function of baseline characteristics of the schools. We found evidence of differential school dropout associated with intervention group, previous school non-participation, percentage of regular smokers at baseline and an interaction between randomization group and baseline level of smoking prevention work. The experimental schools were significantly more likely to stay in the study over time; among them those with high baseline levels of smoking prevention activities had a higher probability of dropping out. The reciprocal of the estimated probability was then used to construct the weights to adjust for school drop-out.

### 4.1. Non-Cumulative Exposure

A first analysis was carried out assuming that the treatment might have had an effect only during the year it was given to the pupils or, in other words, that the exposure did not have a cumulative impact over time. We report only the results obtained after weight adjustment for missing values.

The simple ITT estimate was found to be −0.079 (p=0.3) for year 1, −0.094 (p=0.2) for year 2 and −0.125 (p=0.1) for year 3. The ITT results were therefore unequivocal in suggesting no significant treatment effect but, as we have already pointed out, an ITT analysis delivers biased treatment effect estimates if there are departures from randomization. The multiple-component nature of the ESFA study made it complicated to assess who actually received the whole prevention program. Two contrasting treatment exposure measures were considered: student’s recall of the computer game (variable *game*) and the school contact person’s report on the number of smoking prevention lessons carried out by the school (variable *prevtime*). The first is a binary treatment element which, being collected at an individual level, allows account to be taken for possible students’ absences from school. However, it can be affected by unmeasured factors such as students’ absent-mindedness or poor memory. On the other hand the second exposure indicator, i.e. that reported by each school’s contact person, is a grouped continuous measure on the amount of time spent in lesson-based anti-smoking activities. It is an ordinal variable with 6 categories corresponding to 0, 1, 2 to 3, 4 to 5, 6 to 10 and more than 10 lessons. This seemed likely to be a more reliable measure of treatment receipt, but it is a school-level indicator and as such it did not allow us to identify partial and variable compliance within a school. It may have happened that in the same school some classes and students complied and some others did not. Furthermore, even from participating schools not all the contact teachers provided this information. Data were available on 38 out of 42 schools at baseline, 30 out of 34 at the first intervention year and 22 out of 26 at the second and third years of the intervention. This required a modified set of non-response weights. Information about the last 2 intervention years were collected through the same questionnaire before the end of the study, meaning that the level of exposure reported for year 3 was only partial, i.e. up to the time the questionnaire was completed. As a consequence, for analyses where the pupils’ level of treatment exposure was assessed though the school contact persons’ reports, we focus on years 1 and 2 and ignore year 3 because the exposure measure was incomplete.

An ATT analysis was then performed by using *game* and *prevtime* as two alternative measures of treatment received. Since adding important covariates can make for a more powerful analysis and can further mitigate problems due to selective attrition, we included the student’s sex and previous smoking status as two additional explanatory variables. The results of the ATT analysis of student’s recall of the computer game are reported in Table 1. These suggest significant intervention effects in years 1 and 2. Equivalent results using the teachers’ reports indicator of treatment received are shown in Table 2 and suggest no intervention effect.

However, as mentioned before, the ATT estimator was likely to be a biased estimator of the treatment effect since group provision and receipt of the prevention program can be correlated with features of the school, teacher and individual student. Methods that account for non-compliance were therefore needed. Non-compliance adjustments based on the propensity score approach are in these cases complicated by the presence of time-varying confounders and by the difficulty of controlling for all the possible variables that may have an influence on the selective treatment exposure. For example, if we consider the exposure to the computer game, the probability of a student recalling the treatment may depend on both individual and school characteristics, some un- or imperfectly measured. We found for instance that in experimental schools good students, who might be expected to be more attentive in class, were more likely to recall the treatment. Since it is not possible to be confident in assuming that we are correcting for all the possible confounders, a more reasonable way to proceed is to use the randomised treatment group assignment as an IV to account for both measured and unmeasured confounders. Since the outcome variable (student’s regular smoking status) was dichotomous, this required the IV estimation to be performed in the simultaneous equations framework. When treatment exposure was assessed through the binary report of computer-game use a bivariate probit model was fitted using the command *biprobit* in Stata [25]. Treatment assignment was found to be a strong IV (p<0.001 in each intervention year). Results are displayed in column 3 of Table 1 and show only marginally significant treatment effects in years 2 and 3.

If treatment receipt was measured by the number of smoking prevention lessons, the corresponding IV estimator for the effect of treatment required estimating a bivariate ordinal probit model with 2 categories for the smoking status outcome equation and 5 categories for the endogeneous treatment equation. This was estimated in Stata using *gllamm*. Results are reported in Table 2 and show no evidence of treatment effect. However, treatment assignment was in this case a poor IV (p>0.1 for years 1 and 2), yielding to wide confidence intervals.

### 4.2. Cumulative Exposure

For the computer game the cumulative exposure was obtained by summing the binary variable game over time. In year 1 exposure was binary, i.e. exposed to the computer game (1) or not (0). In year 2 cumulative exposure had 3 categories: (00) = 0, (01 or 10) = 1 and (11) = 2. In year 3 there were four categories: (000) = 0, (100 or 010 or 001)=1, (110 or 101 or 011)=2 and (111)=3. The analysis was then carried out for each of the intervention years by fitting a pair of simultaneous equations formed by a probit model for the binary smoking outcome and an ordinal probit model for the cumulative exposure, where the randomization assignment variable was used as an IV.

For the number of non-smoking lessons reported by the school contact person the computation of the cumulative exposure was not so straightforward because the amount of exposure was sometimes known only up to an interval approximation. School contact persons were asked to choose among 0, 1, 2–3, 4–5, 6–10 and >10 lessons (no school was reported to fall in this last category). The dose of the treatment received by the subject was therefore taken as the midpoint of the interval in which it was known to fall (if for example the school contact person reported a number of lessons between 4 and 5, the midpoint dose was taken as 4.5) and the cumulative exposure was derived by adding these midpoint doses over time.

The resulting analysis required simultaneous equations formed by a probit model for the smoking outcome and a linear model for the approximately continuous cumulative exposure for each of years 1 and 2. Both this and the previous model were fitted in Stata using the *gllamm* procedure. Results are reported in Table 3. Since cumulative exposure is equivalent to the already analyzed current exposure data in year 1, results are shown only for year 2 onwards. Intervention group proved a strong IV for cumulative exposure to the computer game (p<0.001 in both year 2 and year 3). When controlling for smoking at baseline and the sex of the pupil, marginally significant beneficial effects are suggested from cumulative exposure to the computer game. By contrast, intervention group proved to be a poor IV for time on generic smoking prevention (p=0.6) and, after controlling for baseline smoking and the sex of the pupil, there was no suggestion of any treatment effect.

## 5. Discussion

In the last decades there has been a lot of debate on the effectiveness of smoking cessation programs and how this type of interventions should be designed and implemented. Conflicting findings from reviews and meta-analyses have been reported in the literature (see for example [26–28]). Several researchers have highlighted the need for improved methodologies in drug use prevention studies where very often complex statistical issues such as differential attrition and unmeasured confounding are not taken into proper account [29]. This paper has presented the analysis of a complex smoking prevention study and has illustrated IV estimation of treatment effects to tackle non-compliance and measurement error for a range of treatment exposure measures in the presence of clustering and dropout.

Our main findings are both empirical, relating to ESFA treatment efficacy, and methodological, for the design of future studies with these methods of analysis in mind. The empirical findings from this study provide little encouragement for believing that typical poorly resourced school-based interventions can bring about substantial long-lasting reductions in smoking behaviour. In large part this may reflect the difficulty of engaging with students [30]. Sussman and colleagues [27] suggested that, to minimize non-compliance and drop-out, interventions should be as fun as possible and they should include activities such as games and dramatizations. In our study we found that the differences between the control and the experimental schools in terms of students’ recall of the intervention were mostly quite modest, apart from that related to a computer game.

Demonstrating an intervention effect is particularly difficult for dispersed and multi-facetted treatment packages such as ESFA. Analyses for the estimation of the effect of the computer game suggested that among those who recalled the intervention it may have had marginally significant cumulative and non-cumulative effects on the last 2 years of the intervention. When treatment exposure was assessed through the number of smoking prevention lessons carried out by the school no evidence of treatment effect was found, but the randomization indicator proved to be a poor IV leading to wide confidence intervals. For several of the other elements of the treatment package, the materials and strategies were becoming integrated into the standard teaching of many schools. Moreover, in practice merely achieving a statistically identifiable impact on treatment exposure proved to be quite a challenge, but this is a necessary preliminary before an IV estimator can demonstrate an independent effect of treatment in the presence of non-compliance. The problems this presented for evaluation are likely to recur in future studies. Our ability to detect the effectiveness of such elements rests heavily on being able to record student treatment exposure rigorously and is improved where reliable record linkage over occasions of measurement can be maintained. In fact, the ESFA study was designed to test the effect of the treatment package as a whole, and not the individual elements. However, to undertake any analysis other than ITT requires an explicit and reliable measure of exposure to each treatment element. In addition, had the intention been to test the individual elements then more IVs would have been required, typically one for each element. In practice this would have required randomisation to a number of treatment groups, rather than just treatment and control, with each group having a distinct combination of treatment elements.

We introduced the IV estimator from a consideration of a joint model for treatment exposure and outcome. A number of alternative effect estimates have been derived from a “counterfactual” approach by means of an explicit comparison of an estimate of each individual’s observed outcome to the hypothetical outcome had the individual received a treatment regime other than that observed, the so-called counterfactual outcome. These approaches emphasize the various assumptions that must be made in deriving expected outcomes for these counterfactuals and highlight the difference between a local average treatment effect (LATE) that is particular to the treatment participation scheme of the evaluation study and an average treatment effect that would pertain were the whole population induced to participate. IV methods in general make implicit assumptions as to the homogeneity of any treatment effect or, if the effect is heterogeneous, the lack of correlation between the response to treatment and the propensity to receive treatment [31]. In the simple binary treatment case the IV estimator can be defined in terms of counterfactuals [32] and be given a local average treatment effect interpretation [33, 34]. The binary treatment case also allows a categorization of participants into so-called compliers and non-compliers, this distinction being observable only among those randomized to treatment. A comparison of outcome differences for the compliers gives the so-called complier average causal effect (CACE) and can be obtained for example by specifying the compliance status as a latent-class for those not randomized to treatment, yielding an estimate equivalent to LATE.

We have illustrated IV methods in the context of a trial where randomization provided an obvious IV. In other applications the search for an appropriate IV is not trivial. Often it is necessary to draw upon imagination, theory and chance to find IVs, the latter often by exploiting “natural experiments”. For instance, Leigh and Schembri [35] use variation in cigarette price, which can be largely influenced by exogeneously determined tax changes, as an IV in their study of the effects of smoking on physical functional status. Genetic polymorphisms are also now being considered as potential IVs [36]. Readers need to be convinced that the assumptions of the method, notably the exclusion restriction, are met for each specific study.

## Figures and Tables

**Table 1.** Estimates of the treatment effects for the computer game by using ATT and IV methods. Analyses were weighted to account for school dropout and adjusted for covariates sex (0 = male, 1 = female) and prevsmok (1 = the student was a regular smoker the year before, 0 otherwise). Robust standard errors are denoted with SE* and are reported between brackets.

	ATT		IV method	
	Estimate (SE*)	*p-value*	Estimate (SE*)	*p-value*
**year 1**				
treatment (*game*)	−0.232 (0.083)	0.005	−0.067 (0.198)	0.734
Sex	0.204 (0.068)	0.003	0.204 (0.066)	0.002
prevsmok	1.611 (0.103)	0.000	1.616 (0.102)	0.000
intercept	−1.369 (0.061)	0.000	−1.406 (0.072)	0.000
**year 2**				
treatment (*game*)	−0.216 (0.069)	0.002	−0.331 (0.180)	0.066
Sex	0.137 (0.074)	0.066	0.141 (0.076)	0.062
prevsmok	1.696 (0.130)	0.000	1.685 (0.127)	0.000
intercept	−1.158 (0.061)	0.000	−1.131 (0.065)	0.000
**year 3**				
treatment (*game*)	−0.011 (0.095)	0.905	−0.498 (0.274)	0.069
Sex	−0.014 (0.111)	0.902	−0.004 (0.112)	0.969
prevsmok	2.161 (0.104)	0.000	2.126 (0.104)	0.000
intercept	−1.341 (0.092)	0.000	−1.228 (0.084)	0.000

**Table 2.** Estimates of the treatment effects for *prevtime* by using ATT and IV methods. Robust standard errors are denoted with SE* and are reported between brackets.

	ATT		IV method	
	Estimate (SE*)	*p-value*	Estimate (SE*)	*p-value*
**year 1**				
treatment (*prevtime*)	−0.007 (0.055)	0.894	−0.035 (0.212)	0.867
Sex	0.258 (0.087)	0.003	0.258 (0.088)	0.003
prevsmok	1.724 (0.090)	0.000	1.720 (0.096)	0.000
intercept	−1.464 (0.177)	0.000	−1.381 (0.634)	0.029
**year 2**				
treatment (*prevtime*)	−0.048 (0.041)	0.244	−0.311 (0.326)	0.342
Sex	0.149 (0.117)	0.202	0.116 (0.128)	0.363
prevsmok	1.752 (0.147)	0.000	1.740 (0.134)	0.000
intercept	−1.125 (0.119)	0.000	−0.536 (0.772)	0.487

**Table 3.** Estimates of the cumulative treatment effects based on the students’ self-reports (computer game) and the school contact persons’ reports (lesson time). Analyses were adjusted for covariates sex (0 = male, 1 = female) and smok0 (1 = the student was a regular smoker at the baseline, 0 otherwise). Robust standard errors (SE*) are reported between brackets.

	Cumulative exposure			
	Computer game		lesson time	
	Estimate (SE*)	*p-value*	Estimate (SE*)	*p-value*
**year 2**				
intercept	−0.962 (0.058)	0.000	−0.065 (1.151)	0.955
Sex	0.199 (0.065)	0.002	0.269 (0.183)	0.141
smok0	1.166 (0.146)	0.000	1.385 (0.206)	0.000
treatment	−0.138 (0.073)	0.059	−0.095 (0.123)	0.441
**year 3**				
intercept	−0.780 (0.084)	0.000		
Sex	−0.000 (0.111)	0.998		
smok0	1.196 (0.217)	0.000		
treatment	−0.150 (0.096)	0.117		
